# 多孔有机框架材料在真菌毒素分离富集与检测中的研究进展

**DOI:** 10.3724/SP.J.1123.2023.08003

**Published:** 2023-10-08

**Authors:** Wei LIU, Zhiwei XU, Rui WANG, Yu ZHAO, Qiong JIA

**Affiliations:** 1.长春中医药大学药学院, 吉林 长春 130017; 1. College of Pharmacy, Changchun University of Chinese Medicine, Changchun 130017, China; 2.长春中医药大学人参科学研究院, 吉林 长春 130017; 2. Jilin Ginseng Academy, Changchun University of Chinese Medicine, Changchun 130017, China; 3.吉林大学化学学院, 吉林 长春 130012; 3. College of Chemistry, Jilin University, Changchun 130012, China

**Keywords:** 金属有机框架, 共价有机框架, 真菌毒素, 富集, 检测, metal-organic framework (MOF), covalent-organic framework (COF), mycotoxins, enrichment, detection

## Abstract

真菌毒素是真菌产生的一类有毒的次级代谢产物,对人体具有致癌、致畸、致突变等严重危害,已引起世界范围的广泛关注。因此,建立准确、快速、灵敏的真菌毒素检测方法具有非常重要的意义。色谱法是常用的检测真菌毒素的方法,但由于真菌毒素种类繁多,分布范围广泛,样品基质复杂,且各类真菌毒素在实际样品中含量极低,难以对其进行直接分析。因此,发展适宜的样品前处理方法,并用于真菌毒素的高效分离富集是必不可少的步骤。近年来,以金属有机框架(MOF)、共价有机框架(COF)为代表的多孔有机框架材料因具有大的比表面积、高的孔隙率、可调的孔径、多样的框架结构、活性位点分布均匀、结构可修饰等优点被广泛应用于真菌毒素的样品前处理领域。同时,这些优点赋予MOF/COF材料以优异的荧光性质、电化学性质,使其在真菌毒素的分析传感等领域也得到了广泛关注。本文针对近年来MOF/COF材料在真菌毒素分离富集中常用的样品前处理方法(固相萃取、分散固相萃取、磁固相萃取、免疫磁珠分离)中的应用进行了综述。同时,针对MOF/COF材料在真菌毒素荧光传感、电化学传感中的研究进行了总结。最后,对存在的问题及未来的发展趋势进行了讨论与展望,为进一步探索MOF/COF材料在真菌毒素中的应用提供参考。

## Abstract (en)

Mycotoxins are a class of toxic secondary metabolites produced by fungi. These substances are carcinogenic, teratogenic, and mutagenic, and cause serious harm to the human body; thus, they have attracted wide attention worldwide. Establishing accurate, rapid, and sensitive methods for the detection of mycotoxins is of great significance. Chromatography is a commonly used technology for mycotoxin detection. However, it is challenging to use in the direct analysis of these metabolites because of the wide variety and distribution of mycotoxins, their complex sample matrix, and their very low content in actual samples. Therefore, the development of suitable sample pretreatment methods for the efficient separation and enrichment of mycotoxins is necessary. In recent years, porous organic framework materials, which are represented by metal-organic frameworks (MOFs) and covalent-organic frameworks (COFs), have been widely applied in the sample pretreatment of mycotoxins owing to their many advantages, which include a large specific surface area, high porosity, adjustable pore size, diverse frame structures, uniform active site distribution, and modifiable structures. In addition, MOF/COF materials feature excellent fluorescence and electrochemical properties, rendering them highly suitable for mycotoxin analysis and sensing. In this article, the recent applications of MOF/COF materials in conventional sample pretreatment methods (e. g., solid-phase extraction, dispersive solid-phase extraction, magnetic solid-phase extraction, and immunomagnetic bead separation) for mycotoxin separation and enrichment are reviewed. Research on the use of MOF/COF materials for the fluorescence and electrochemical sensing of mycotoxins is also summarized. Finally, the existing challenges and future development trends of these materials are discussed and prospected to provide a reference for future research on the applications of MOF/COF materials in mycotoxin detection and analysis.

真菌毒素(mycotoxin),也称霉菌毒素,是由真菌产生的一类有毒的次生代谢产物,对肝脏、肾脏、造血系统、免疫系统和生殖系统有严重的毒性以及致癌、致畸、致突变等作用^[1]^。真菌毒素具有种类繁多、来源广泛等特点,迄今为止,已发现500多种化学结构不同的真菌毒素,其中黄曲霉素(AFs)、赭曲霉素A(OTA)、玉米赤霉烯酮(ZEN)、棒曲霉素(PAT)、3-硝基丙酸(3-NPA)等因具有较强的毒性,受到了广泛的关注。最易受真菌毒素污染的作物是谷物,尤其是小麦、玉米、大麦、黑麦、燕麦等^[2]^。另外,中药材在采收、加工、贮存等过程中若处理不当,也很容易被真菌毒素污染。真菌毒素污染问题已经成为中药材生产和使用过程中的重要安全性问题之一^[3]^。

在已报道的真菌毒素的检测分析方法中,酶联免疫吸附测定法应用较广,但该方法需依赖昂贵的抗体和酶,具有制备成本高、耐酸碱性低、稳定性差、储存不便、操作步骤繁琐等缺陷,因此很难满足食品安全中快速检测的要求。色谱法是另一种常用的真菌毒素的检测方法,然而,真菌毒素种类繁多、分布范围广泛、样品基质复杂,且各类真菌毒素在实际样品中含量极低,这些条件阻碍了色谱法在真菌毒素检测中的直接应用。因此,在色谱分析前开发高效的样品前处理方法十分重要。

目前,已有一些吸附材料被用于真菌毒素的分离富集,如超交联聚合物^[4]^、纳米复合材料^[5]^、分子印迹聚合物^[6]^、多孔有机框架材料^[7]^等。其中,多孔有机框架材料具有比表面积大、孔径可调节、活性位点分布均匀、结构可修饰等优点,在气体储存^[8]^、吸附分离^[9,10]^、化学催化^[11]^、化学传感^[12]^等方面均得到了应用。金属有机框架(MOF)和共价有机框架(COF)是多孔有机框架材料最典型的代表,在真菌毒素的分离富集领域已受到广泛关注。同时,MOF/COF材料的结构特性使得其具有优异的荧光和电化学性质,基于MOF/COF的荧光及电化学传感器由于操作简单、响应迅速、灵敏度高,已被广泛用于真菌毒素的分析传感检测。

目前,已有一些综述文章分别针对MOF或COF材料在真菌毒素样品前处理或分析传感中的研究进行了总结^[7,13⇓-15]^。例如,Xin等^[7]^综述了COF吸附剂在食品污染物(包括多环芳烃、生物胺、杀虫剂、重金属离子、非法添加剂、生物毒素等)富集分析中的应用;林志琦等^[14]^总结了MOF传感器在真菌毒素检测中的研究进展。本文系统综述了MOF和COF这两类多孔有机框架材料在真菌毒素分析检测中的研究应用,涵盖了真菌毒素色谱分析中的样品前处理方法(固相萃取(SPE)、分散固相萃取(DSPE)、磁固相萃取(MSPE)、免疫磁珠分离(IMS))及得到了广泛关注的荧光/电化学传感研究的应用进展。

## 1 多孔有机框架材料概述

多孔有机框架材料是多孔材料的一种,近年来得到了快速的发展,目前以MOF、COF为代表的多孔有机框架材料在样品前处理及分析传感等领域都得到了广泛的应用。MOF是一类通过配位键将过渡金属离子和有机配体连接起来,经过一系列自组装行为形成的多孔材料。根据采用的金属和有机配体不同,可形成多种拓扑结构。COF的骨架是由纯有机构筑单元通过有机官能团反应桥联搭建的,完全由C、H、O、N、B等轻元素通过共价键组成,所以密度较小,化学稳定性和热稳定性更好。因为有机化学反应类型种类繁多,构筑单元多种多样,所以理论上来讲可以合成的COF是层出不穷的。

MOF/COF材料的合成主要通过将前驱体置于反应容器中,外加能量促进结晶。二者的合成方法有很多种,其中电化学合成法^[16]^、微波合成法^[17]^、光化学合成法^[18]^、辐射化学合成法^[19]^等合成方法尚未得到广泛应用,还需要深入研究结晶过程和机理;常见的合成方法有水/溶剂热合成法^[20⇓⇓⇓⇓⇓⇓⇓⇓⇓-30]^、室温搅拌合成法^[31⇓⇓⇓-35]^、超声合成法^[36]^,这些合成方法各有优缺点,具体见表1。

**表 1:** MOF和COF的常用合成方法

Preparation method	Advantages	Disadvantages	Ref.
Water/solvent thermal synthesis	high purity, high crystallinity, regular shape	requirement of high temperature or high pressure	[20-30]
Room temperature agitation synthesis	simple operation, fast synthesis, high crystallisation rate and yields	poor crystallinity	[31-35]
Ultrasonic synthesis	simple equipment, fast reaction speed, low energy consumption	poor purity	[36]

## 2 多孔有机框架材料在真菌毒素分离富集中的应用

MOF/COF材料由于独特的结构与性质,被广泛用于气体、金属离子、有机小分子、生物大分子等的吸附中。由MOF/COF材料构建的吸附剂,可以通过配位、*π-π*、氢键、疏水作用等多种作用力与真菌毒素相互作用,因此其在真菌毒素分离富集中的应用引起了研究者的广泛关注。目前,MOF/COF分离富集材料在真菌毒素样品前处理中的应用技术主要包括SPE、DSPE、MSPE、IMS等。

### 2.1 固相萃取

SPE是通过吸附剂选择性吸附目标物,再采用溶剂解吸目标物,以达到分离富集的目的。该技术具有富集效率高、选择性较好、溶剂用量少等优点^[37,38]^。MOF/COF材料已被广泛用作真菌毒素的SPE吸附剂。例如,Du等^[39]^建立了一种多目标、高效率、低成本的去除植物油中真菌毒素的方法。作者以MOF-235为吸附剂,在30 min内即可同时去除超过96.1%的AFs和83.3%的ZEN,而且,用MOF-235处理的植物油表现出较小的细胞毒性。MOF-235具有足够的去除目标残留物的能力以及安全性和可重复使用性,可用于去除受污染植物油中的多种真菌毒素。

MOF除了直接作为吸附剂用于富集外,还可以作为优良的模板用来制备具有中空结构的吸附材料,中空结构中存在的较大空腔使得合成的材料具有更大的比表面积、丰富的孔隙率、化学稳定性、表面渗透性及低密度等优点。Yang等^[40]^利用ZIF-8作为自牺牲模板,制备了一种具有中空结构的COF材料(HCOF)。作者首先合成了核壳结构的ZIF-8@COF,然后将ZIF-8@COF溶解在醋酸溶液中去除ZIF-8以得到HCOF。与传统方法合成的COF相比,HCOF具有更高的稳定性、通用性及对AFs更高的吸附性能。以合成的HCOF为固相萃取吸附剂,与HPLC-MS联用,成功用于婴幼儿奶粉中AFs的检测。

为了提高复杂基质中待分析物质的选择性,分子印迹聚合物(MIPs)常被用作SPE吸附剂^[41,42]^。Huang等^[43]^合成了基于MIL-101的表面分子印迹聚合物(MIL-101@MIPs),结合HPLC用于检测谷物中的ZEN。Fatemeh等^[44]^利用深共晶溶剂技术与分子印迹技术相结合,制备了基于MOF的AFs分子印迹聚合物,用作SPE吸附剂,成功实现了对谷物样品中4种AFs的富集。Li等^[45]^报道了一种在室温下简单合成的具有强疏水性的纳米花COF,据此制备了SPE吸附剂COF@MIP,建立了SPE与HPLC联用法,并用于5种常见谷物中AFs的检测。该方法在痕量真菌毒素的预处理和检测方面具有潜在的应用价值。

### 2.2 分散固相萃取

DSPE是一种将固体吸附剂直接添加到样品溶液中,待吸附剂和分析物之间相互作用后,通过离心实现目标物快速分离富集的一种方法。DSPE克服了SPE由于扩散和传质速率限制而柱平衡时间较长的缺点,具有时间更短、成本更低、操作更简单、有机溶剂消耗更少等优点。Mohebbi等^[46]^合成了维生素B_3_-MOF,作为分散固相萃取的吸附剂,结合HPLC-MS检测,用于提取和富集豆奶样品中的AFs。该吸附剂是以维生素B_3_为连接剂,水为反应溶剂,在温和的条件下绿色合成得到的。在整个萃取过程中,吸附剂和有机溶剂的用量较小,对环境友好。另外,Rezaei等^[47]^采用水热法合成了Cu/Ni双金属MOF材料并用作DSPE吸附剂,结合HPLC-荧光检测,成功用于水样和大米中4种AFs(AFB_1_、AFB_2_、AFG_1_、AFG_2_)的检测,定量限为0.04~0.15 ng/mL。

### 2.3 磁固相萃取

MSPE是以磁性材料作为吸附剂的一种分散固相萃取技术。由于在外加磁场下即可实现磁性吸附剂与分析物的迅速分离,与常规的SPE相比,MSPE具有操作简单、省时快速等优势^[48,49]^。因此,MSPE在真菌毒素分离富集中的应用已见诸多报道,其中,对AFs的分离富集受到了最广泛的关注。例如,Li等^[25]^以磁性金属有机框架(Mg/Zn-MOF-74@Fe_3_O_4_ MNPs)为吸附剂,建立了AFB_1_的检测方法。该吸附剂对AFB_1_表现出优异的吸附性能,能够快速富集和分离复杂基质中的AFB_1_,并且合成条件简单温和,价格低廉,因此有望成为商用免疫亲和柱的替代品,具有广阔的应用前景。Durmus等^[50]^采用超声搅拌共沉淀法制备了磁性纳米颗粒MOF复合材料(MIL-53(Al)-SiO_2_@Fe_3_O_4_),将其作为磁性固相萃取吸附剂,用以检测冬季凉茶中的AFB_1_。Ghorbani等^[51]^制备了一种新型、低成本的吸附剂Fe/Ni-MIL-53@chitosan@Fe_3_O_4_,分别用于磁固相萃取中药材蒸馏物、食品和实际水样中的4种AFs。该吸附剂由壳聚糖、磁性Fe_3_O_4_纳米颗粒和双金属MOF依次通过水热合成法和化学合成法制备而成,Fe/Ni-MIL-53提高了AFs的萃取效率,壳聚糖增加了该吸附剂的稳定性,有效避免了吸附剂在超声条件下被破坏。

Li等^[52]^以2,5-二羟基-1,4-苯二甲醛(Dt)和4',5'-双(4-氨基苯基)-[1,1:2',1″-三联苯]-4,4″-二胺(BAPTPDA)为单体合成了一种磁性COF吸附剂。该吸附剂表现出对黄曲霉素M_1_(AFM_1_)、M_2_(AFM_2_)优异的吸附性能,结合HPLC-MS检测,实现了牛奶中AFM_1_、AFM_2_的快速检测,该方法线性范围宽(0.01~100 μg/kg)、检出限低(0.0069~0.0078 μg/kg)。同年,该课题组^[53]^采用1,2,4,5-四(4-甲酰基苯基)苯(TFPB)和对苯二胺(PPD)这两种新型单体,在室温下制备了一种磁性固相萃取材料M-COF,并与HPLC-MS联用,用于测定食品(牛奶、食用油、大米)中的4种AFs。该M-COF对AFs的吸附容量为69.5~92.2 mg/g、检出限为0.01~0.05 μg/kg,且提取过程简单、快速,可重复使用8次以上。

除AFs外,MSPE对OTA的分离富集也已被报道。Wei等^[54]^制备了一种磁性MOF(UiO-66-NH_2_),该材料具有合成简单、操作方便、性能优异的特点,10 min内对OTA的吸附效率高达94%。在30 min即可完成检测,线性范围较宽(0.1~100 ng/mL),检出限较低(0.28 ng/mL)。另外,Yang等^[55]^制备了一种磁性共价有机框架材料(Fe_3_O_4_@COF),并成功应用于啤酒、白酒和醋中OTA的富集和分析。由于COF本身具有较大的比表面积和高孔隙率,以及COF与OTA之间的氢键、*π-π*、疏水等相互作用,采用Fe_3_O_4_@COF成功实现了对OTA的高选择性富集。

针对不同种类真菌毒素的混合体系,研究者们也进行了大量的工作。Wei等^[31]^以核壳结构的磁性共价有机框架(Fe_3_O_4_/COF-TpBD)为吸附剂,构建了一种简单、快速、灵敏、涡流辅助的MSPE方法,结合HPLC-MS用于玉米中10种真菌毒素(包括AFs、OTA、镰孢菌素等)的同时检测,线性范围为0.05~50 μg/kg,检出限为0.02~1.67 μg/kg。Wang等^[30]^以磁性COF(TAPT-DHTA)纳米复合材料作为MSPE吸附剂,结合UHPLC-MS用于水果中9种真菌毒素的检测。该复合材料以Fe_3_O_4_为磁芯,1,3,5-三-(4-氨基苯基)三嗪(TAPT)和2,5-二羟基对苯二甲醛(DHTA)为两个构建单元,通过简单的模板沉淀聚合法制备而成。由于具有丰富的羟基和芳香环,该复合材料能有效捕获真菌毒素,所建立的方法线性范围较宽(0.05~200 μg/kg),检出限低(0.01~0.5 μg/kg)。Guo等^[56]^以MIL-101(Cr)@Fe_3_O_4_纳米复合材料为吸附剂,开发了一种高效快速的MSPE方法,结合UHPLC-MS成功用于多种农产品中9种真菌毒素的检测,该方法灵敏度较高,定量限范围为0.08~0.20 μg/kg。与传统的SPE相比,该方法具有简便、省时、对环境友好的特点。

### 2.4 免疫磁珠分离

IMS是通过纳米磁珠表面修饰的不同官能团与抗体特异性结合,将目标物从样品中识别和富集出来的一种样品前处理技术。目前,将IMS用于真菌毒素分离富集的文献报道较少。Han等^[57]^制备了一种基于MOF复合材料的免疫磁珠,成功用于富集脱氧雪腐镰刀菌烯醇(DON)、ZEN、T-2毒素、HT-2真菌毒素。每100 mg复合物的最大毒素吸附量为DON 688.26 ng、ZEN 864.98 ng、T-2/HT-2 2801.80 ng,且能够保持较高的使用稳定性。另外,该工作对所合成的免疫磁珠的富集效果进行了评价,即使用该免疫磁珠和商用的DZTMS-PREP免疫亲和柱同时处理玉米、小麦、燕麦粉3种样品,实验结果显示,两种处理方法对4种真菌毒素的富集结果一致。

## 3 多孔有机框架材料在真菌毒素分析传感中的应用

MOF/COF具有结构可调、孔隙率高、易于功能化及良好的光电效应等优点,被广泛用于真菌毒素传感器的构建。目前,MOF/COF在真菌毒素分析传感中的应用主要集中于荧光传感和电化学传感(表2)。

**表 2:** MOF/COF在真菌毒素分析传感中的应用

Sensor	Detection method	Analyte	Real samples	LOD/(ng/L)	Ref.
NH_2_-MIL-53(Al)	fluorescence sensing	AFB_1_	tea	1.167×10^4^	[33]
COFs TpBD	electrochemical sensing	AFB_1_	milk	150	[36]
Mn^2+^@UIO-66(Zr)-(COOH)_2_	electrochemical sensing	OTA	corn, orange juice	0.289	[58]
AuNPs@Cd-MOF-74	electrochemical sensing	OTA	red wine	10	[59]
Co/NCNT-Ab_2_	colorimetric-fluorescence	OTA	corn, millet	0.21	[60]
	dual-mode sensing			0.17	
UiO-66	electrochemical sensing	OTA	red wine	7.9×10^-11*^	[61]
Dpy-NhBt-COF@Tb^3+^	fluorescence sensing	OTA	wheat	1.35×10^-2*^	[62]
COF-Au-MB-Apt	electrochemical sensing	OTA	-	0.12	[63]
Co/Fe-MOFs	electrochemical sensing	OTA	-	3.0×10^-4^	[64]
Ag NPs@ZnMOF@MIP	fluorescence sensing	PAT	environmental water, apple juice	0.06^*^	[23]
MIP/Au@PANI/SeS_2_@Co MOF	electrochemical sensing	PAT	apple juice	6.6×10^-7*^	[27]
Zr-MOFmix	fluorescence sensing	PAT	apple juice	0.871	[65]
Pt@AuNRs/Fe-MOFs/PEIrGO	electrochemical sensing	PAT	apple juice, apple wine	4.14×10^-2^	[66]
AuNPs@Ce-TpBpy COF	electrochemical sensing	ZEN	cornflour	0.389	[67]
ZrMOF@MPDB	fluorescence sensing	3-NPA	sugarcane juice	15^*^	[26]
Zn-CoMOF/Ti_3_C_2_ MXene/Fe_3_O_4_-MGO	electrochemical sensing	MPA	grass silages	2.1×10^-2*^	[32]
Hemin@UiO-66-NH_2_	electrochemical sensing	FB_1_	maize	24	[34]

* Unit, μmol/L. AFB_1_: aflatoxin B_1_; OTA: ochratoxin A; PAT: patulin; ZEN: zearalenone; 3-NPA: nitropropionic acid; MPA: mycophenolic acid; FB_1_: fumonisin B_1_.

### 3.1 赭曲霉素A

OTA又名甲酸异香豆素,于20世纪70年代从赭曲霉菌中分离得到,是一种毒性最强的赭曲毒素,具有免疫毒性、肝肾毒性、致畸性、致突变性和致癌性^[68]^。基于MOF/COF的荧光传感器、电化学传感器已被诸多研究者开发并用于OTA的分析传感。如Liu课题组^[60]^以ZIF-8为前驱体,通过高温热解ZIF-8@ZIF-67,制备了具有中空核壳结构的氮纳米管掺杂MOF衍生多孔材料(Co/NCNT),并基于Co/NCNT的氧化酶活性,构建了比色-荧光双模式传感器用于检测OTA(图1),该方法的线性检测范围为0.001~10 μg/L,检出限低至0.21 ng/L(比色法)和0.17 ng/L(荧光法)。随后,该课题组^[69]^以Cu_2_O为模板合成了CuFe-MOF,并进一步通过高温热解制备了Cu@Fe-NC,构建了比色法和比率荧光法的双模式传感器,成功实现了OTA的测定,线性范围为0.001~10 μg/L,检出限为0.52 ng/L。

**图 1:**
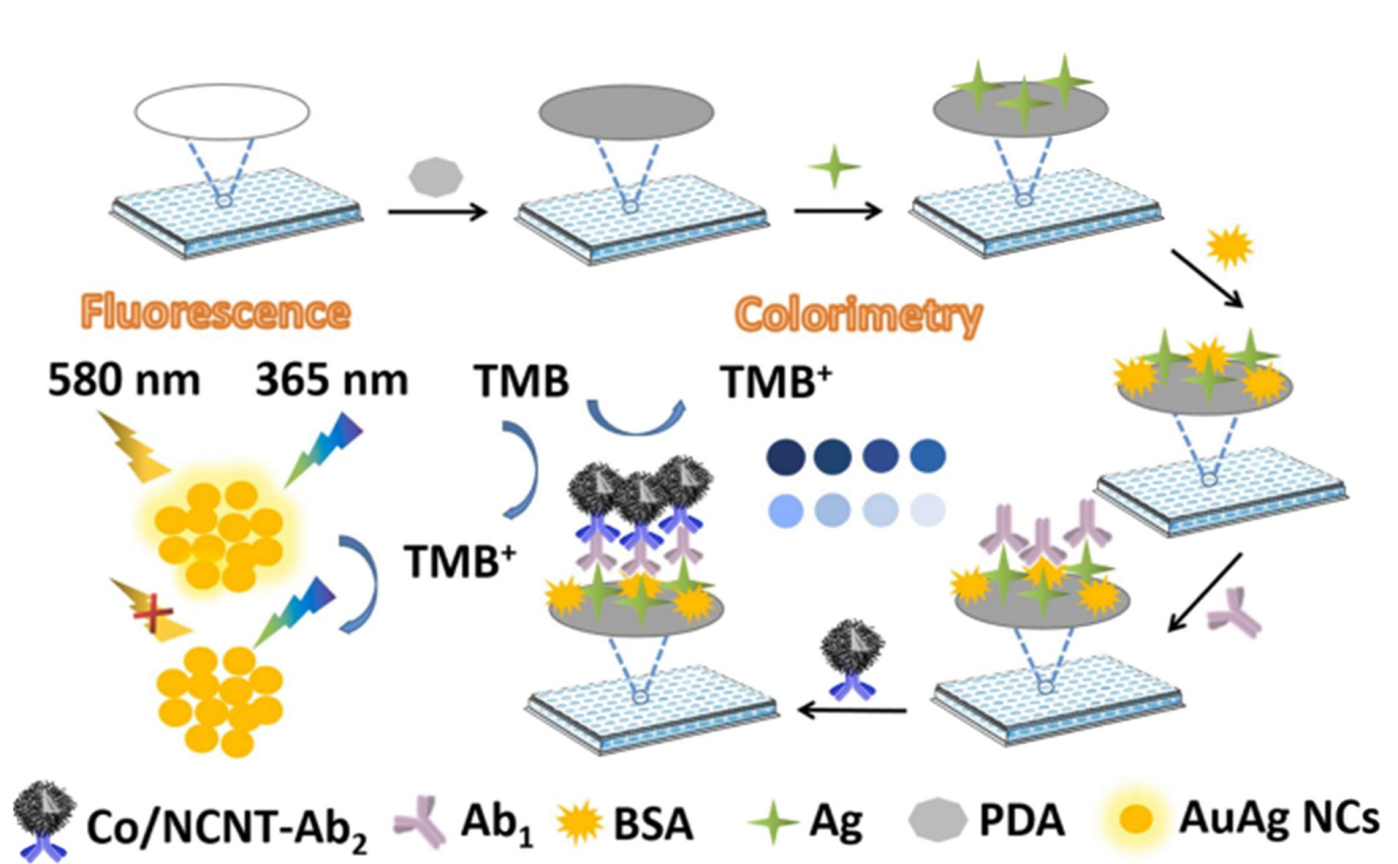
Co/NCNT作为氧化酶模拟物的双模式免疫传感器示意图^[60]^

MOF/COF在OTA的电化学分析传感领域的应用也已见报道。Zhou等^[63]^在热调节丝网印刷电极模块上构建了一种适配体传感器(COF-Au-MB-Apt),成功实现了对谷物样品中OTA的检测,该方法线性范围较宽(2.0×10^-7^~0.6 μg/mL),检出限较低(0.12 pg/mL)。李梅等^[70]^以负载铂纳米颗粒的金属有机骨架纳米酶(PtNPs@Mn-MOF)为电极基底,构建了一种无标记型电化学适配体传感器,用于OTA的定量检测。以2,5-二羟基对苯二甲酸为配体,采用水热合成法制备了具有良好过氧化物酶活性的Mn-MOF-74,随后将铂纳米颗粒负载在Mn-MOF-74上,制备得到了PtNPs@Mn-MOF纳米复合材料。该生物传感器采用的U盘式小型电化学工作站小巧方便、易于携带,可用于实际样品中OTA的现场检测,为食品安全检测提供了一种新方法。最近,Guan等^[71]^报道了一种基于DNA步行器的双信号比率型电化学适配体传感器,用于红酒样品中OTA含量的测定,并将所测结果与商业的酶联免疫试剂盒进行比较,结果令人满意。可以预期,基于MOF/COF的荧光、电化学传感器将在OTA的分析传感中得到越来越广泛的关注。

### 3.2 黄曲霉素

AFs是二氢呋喃香豆素的衍生物,是所有已知的真菌毒素中对食品和饲料污染最严重、关注度最高、研究最广泛的毒素。AFB_1_是AFs中毒性最强的一种毒素,具有致癌性和致畸性,被国际癌症研究机构划分为Ⅰ类致癌物^[36]^。Hu等^[20]^设计合成了一种基于MOF的LMOF-241荧光传感器,用于AFB_1_的高选择性检测。合成的荧光传感材料LMOF-241发蓝绿色光,具有极高的量子产率(92.7%),是迄今为止性能最好的荧光传感器之一。作者通过研究LMOF-241对AFB_1_的传感机理发现,AFB_1_对LMOF-241产生的淬灭原因是电子转移所致而非能量转移。Wang等^[33]^构建了一种基于超低浓度铝金属有机框架的开启式荧光传感平台,用于检测茶叶样品中的AFB_1_(图2)。将NH_2_-MIL-53(Al)作为荧光传感平台,是因为其在水溶液中具有呼吸效应和稳定性。此外,氨基作为活性基团可以与AFB_1_相互作用,两者之间的作用力表现为氢键、配位键和酸碱效应。

**图 2:**
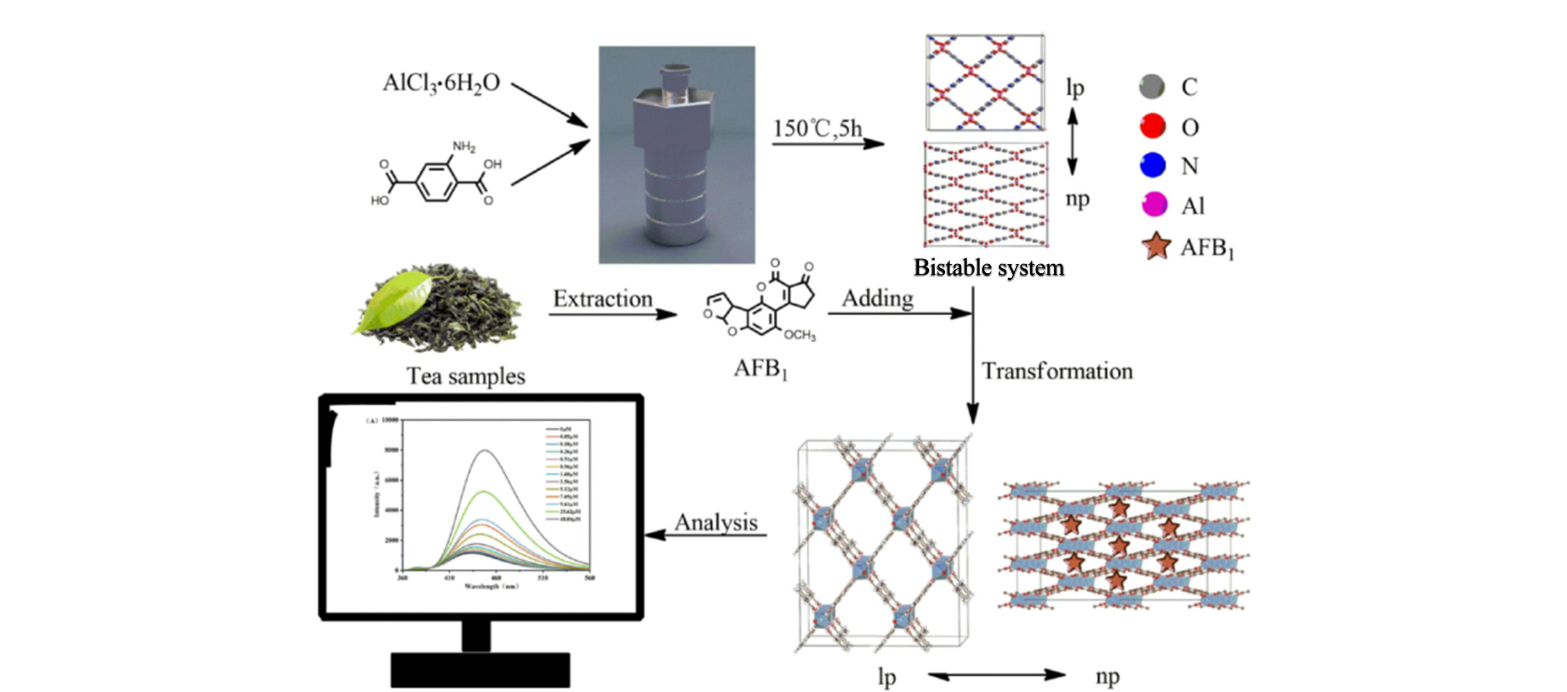
基于荧光MOF的AFB_1_荧光检测示意图^[33]^

除了上述荧光传感器,基于MOF/COF的电化学传感器因选择性好、操作简单快捷等特点也被用于AFs的传感。Pang课题组^[29]^构建了COF(TpBD)修饰的玻碳电极,并结合滚环扩增(RCA),设计了一种电化学-酶联免疫传感器(EC-ELISA),用于乳制品中AFM_1_的检测。该方法的线性范围为0.5~80 ng/mL,检出限为0.15 ng/mL。作者将COF与核酸信号放大相结合,极大地提高了AFM_1_的灵敏度,该方法也为其他生物大分子的检测提供了新思路。随后,该课题组又通过TpBD在电极表面原位生长构建了一种电化学传感器,同样用于AFM_1_的检测^[36]^。TpBD具有较大的表面积,且TpBD与信号探针之间具有较强的*π-π*作用。该方法的线性范围为0.5~80 ng/mL,检出限为0.15 ng/mL。Liao等^[35]^利用仿生矿化MOF封装了大量琥珀酰化辣根过氧化物酶(sHRP),设计了一种电化学免疫传感器。仿生矿化MOF不仅保护sHRP不变性,还促进信号的传输,可提供比常规sHRP偶联物更强的检测信号,实现对痕量AFB_1_的检测。该方法具有线性范围宽(5.0×10^-5^~10 ng/mL)、检出限低(20 fg/mL)的特点,并具有较好的稳定性、特异性和重现性。

### 3.3 3-硝基丙酸

3-硝基丙酸(3-NPA)是曲霉属和青霉属的少数菌种产生的有毒代谢产物,广泛存在于变质的甘蔗中,中毒的主要表现为中枢神经系统受损^[72]^。3-NPA具有很强的酸性,荧光激发和发射波长会随pH变化发生改变,据此,研究者设计了多种用于3-NPA分析传感的MOF荧光探针材料。例如,Guo等^[26]^报道了一种稳定的纳米级单激发比率荧光pH传感器。在2.5~8.6的pH值范围内,作者通过后修饰策略,将萘二甲酰亚胺对锆基金属有机框架(Zr-MOF)进行衍生化处理,制备得到荧光传感材料(ZrMOF@MPDB)。该传感器对pH值具有快速、灵敏和线性响应,并可成功用于甘蔗汁中3-NPA的检测,检出限低至15 μmol/L。Zhao等^[28]^构建了一种基于双发光MOF(Eu-MOF)的pH调节比率荧光传感器,用于甘蔗中3-NPA的检测。在pH值3.0~4.0范围内,该传感器显示出比率发光特性。作者使用基于Eu-MOF的试纸,实现了对3-NPA的快速、可视化和高灵敏度检测。此外,Tian等^[73]^合成了一种基于Cd(Ⅱ)的微孔发光MOF,并通过客体吸附将异硫氰酸荧光素染料分子固定在MOF中,实现了对3-NPA的有效传感,检出限低至0.135 mol/L(图3)。

**图 3:**
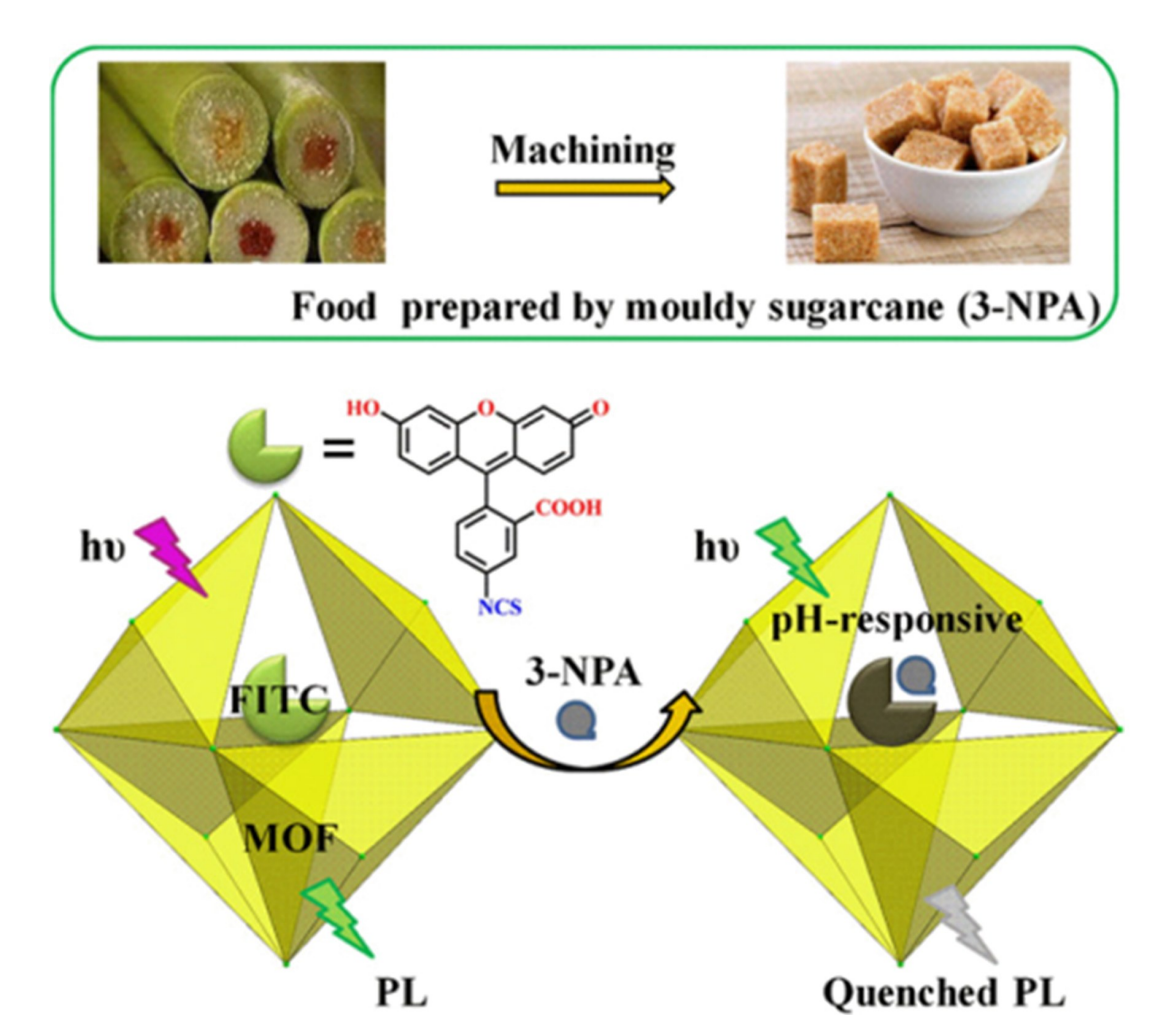
基于Cd(Ⅱ)的MOF复合材料对3-NPA的荧光传感示意图^[73]^

### 3.4 玉米赤霉烯酮

ZEN又名F-2毒素,是一种2,4-二羟基苯甲酸内酯类化合物,具有生殖毒性、免疫毒性、肝肾毒性^[74]^。Chen等^[67]^提出了一种基于便携式电化学工作站的适配体传感器,用于ZEN的定量检测。首先,作者用铈对TpBpy COF进行改性,制备含铈的共价有机骨架材料(Ce-TpBpy COF),随后与金纳米粒子AuNPs结合生成了复合材料(AuNPs@Ce-TpBpy COF),最后借助丝网印刷电极和U盘电化学工作站构建了用于ZEN的电化学传感器。采用计时电流法于Tris-醋酸缓冲溶液中测定AuNPs@Ce-TpBpy COF对过氧化氢还原的催化电流,当体系中存在ZEN时,ZEN与适配体特异性结合,阻碍了电子的传递,使电极表面修饰的AuNPs@Ce-TpBpy COF对过氧化氢还原的催化电流变小,通过催化电流的减小值(Δ*I*)实现了对ZEN的定量检测。

中药材薏苡仁因淀粉及多糖的含量较高,极易受真菌毒素ZEN的污染。Lai等^[24]^报道了一种用于电化学适配体的传感材料P-Ce-MOF@MWCNTs,该材料通过将聚乙烯亚胺对铈基MOF和多壁碳纳米管纳米复合材料进行功能化制备而成,具有较高的比表面积和优异的电化学活性。作者通过电沉积法将甲苯胺蓝(TB)沉淀在合成的复合材料上,并滴入铂@金纳米颗粒(Pt@Au)用以附着ZEN适配体,构建了电化学适配体传感器,并成功用于中药材薏苡仁中ZEN的检测(图4)。

**图 4:**
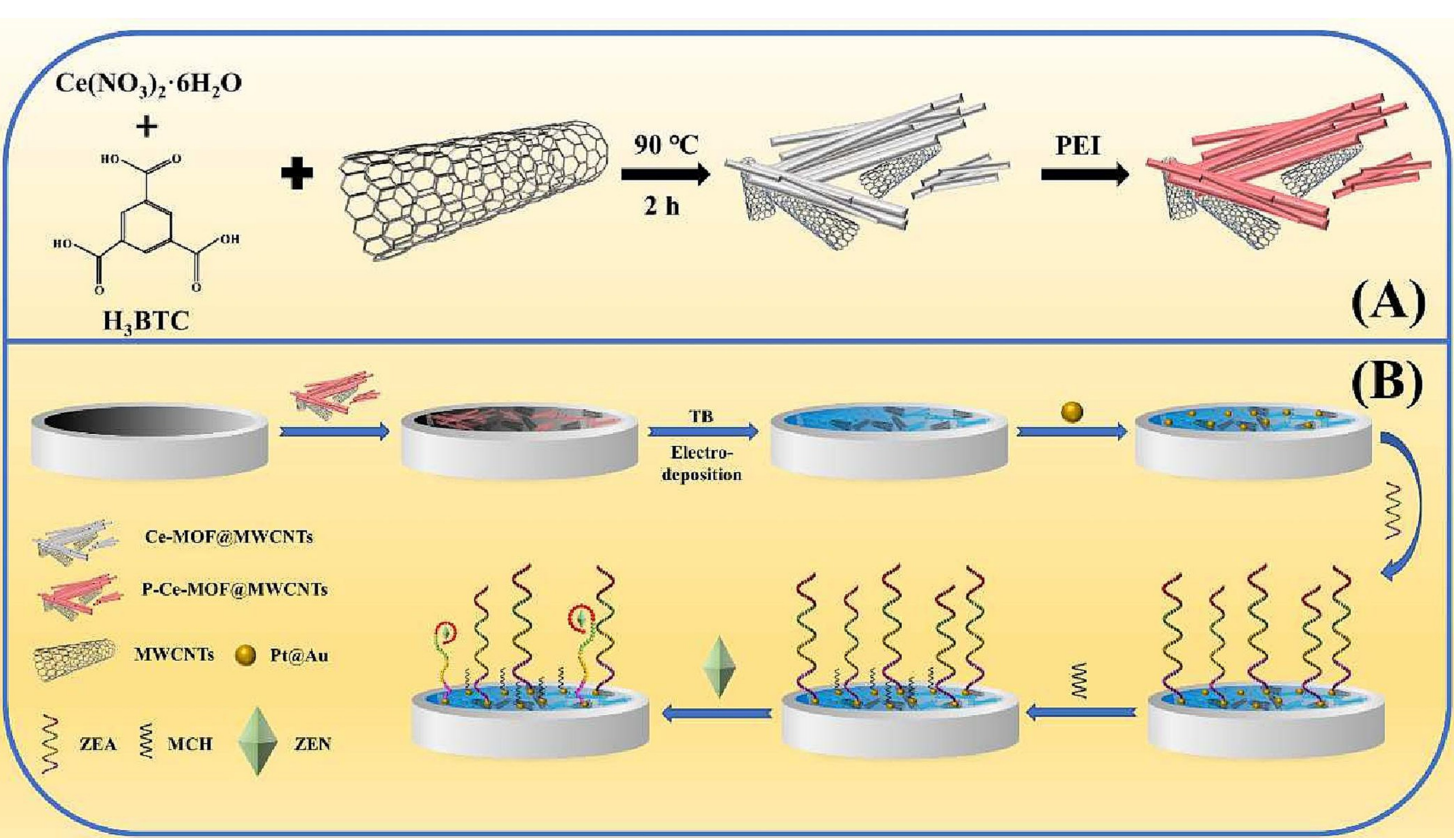
(A)P-Ce-MOF@MWCNTs的合成与(B)电化学适配体传感器的构建示意图^[24]^

### 3.5 其他真菌毒素

真菌毒素种类繁多,MOF/COF除了用于构建OTA、AFs、3-NPA、ZEN 4种真菌毒素的传感器外,在其他真菌毒素如桔青霉素、T-2毒素、PAT、伏马毒素(FB)、DON等传感中的应用也被广泛研究。桔青霉素又名桔霉素,广泛污染玉米、大米等农作物,对人类及动物具有严重的致畸、致癌和诱发基因突变等作用^[75]^。Hitabatuma等^[21]^构建了一种基于MOF(UiO-66)的荧光传感器,用于检测食品和饲料中的桔青霉素。以苯甲酸和对苯二甲酸作为混合配体合成了具有稳定荧光的UiO-66,合成的UiO-66具有较大的比表面积(1390 cm^2^/g),对桔霉素的吸附容量高达150 mg/g,吸附效率达80%。该传感器成功实现了小麦和饲料样品中桔青霉素的检测。

T-2毒素是一种镰刀菌毒素,是农产品中常见的污染物,是一种公认的真核蛋白合成抑制剂,可对皮肤、肾脏、肝脏、大脑、造血、淋巴、胃肠道和生殖系统等多个器官造成损伤^[76]^。Zhao等^[22]^筛选出一种淬灭效率高的NH_2_-UiO-66,采用Cy3标记的适配体(Cy3-aptamer)构建了一种荧光传感器,用于T-2毒素的检测。借助*π-π*、氢键、配位作用,NH_2_-UiO-66可以吸附并淬灭Cy3-aptamer发出的荧光,在T-2毒素存在的情况下,能够识别并结合Cy3-aptamer,导致NH_2_-UiO-66/Cy3-aptamer复合物解体,致使能量转移过程被阻断,荧光强度得以恢复,从而实现对T-2毒素的高灵敏度检测(图5)。

**图 5:**
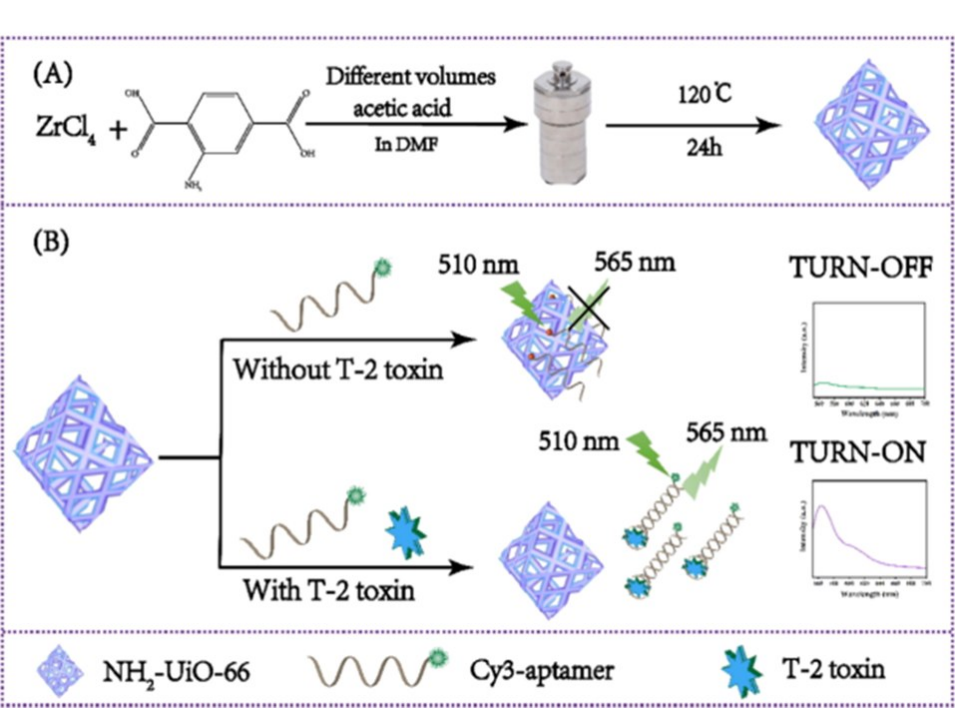
Cy3-aptamer/MOF荧光适配体传感器的原理示意图^[22]^

PAT又称展青霉素,普遍存在于腐烂的苹果及苹果汁、苹果酒等苹果制品中,对胃肠、肝肾及免疫系统具有明显毒性^[66]^。Yan等^[77]^基于催化发夹自组装策略,将硫量子点(SQDs)封装在MOF-5-NH_2_中,构建了一种荧光传感器SQDs@MOF-5-NH_2_,用于检测苹果汁中的PAT。与MOF-5-NH_2_相比,SQDs@MOF-5-NH_2_具有更强的荧光强度和良好的水溶性,其实验结果与HPLC的测定结果一致,证明该荧光传感器具有良好的实际应用价值。

伏马毒素是一种由串珠镰刀菌产生的水溶性代谢产物,主要污染玉米、小麦、高粱、水稻等粮食,迄今为止,伏马毒素已被发现的种类至少有15种,其中伏马毒素B_1_(FB_1_)的毒性最强,污染最广泛。FB_1_能够引起细胞膜脂质改变、鞘脂代谢紊乱、细胞氧化损伤、细胞凋亡、细胞自噬等,甚至有致癌的潜在威胁^[78]^。Sun等^[34]^设计合成了一种DNA水凝胶包覆MOF的刺激响应型电化学传感器,用于检测食品样品中的FB_1_。DNA-聚丙烯酰胺水凝胶适配体传感器是通过杂交链式反应在MOF骨架上形成的,该传感器对FB_1_具有明显的刺激性响应,在对溶液及食品样品中的FB_1_进行检测时,表现出较好的分析性能,该方法的线性范围为0.05~100 ng/mL,检出限为0.024 ng/mL。

DON又名瓜萎镰菌醇,常存在于被污染的粮食中,食用过量DON的人或动物会引起肠胃炎,长期食用会导致发育和繁殖受阻^[79]^。Yu等^[80]^构建了一种荧光-表面增强拉曼散射双模式(FL-SERS)适配体传感器,成功用于DON的高灵敏检测,该方法的检出限为0.08 ng/mL(FL)和0.06 ng/mL(SERS)。除了以上真菌毒素,可以预期,基于MOF/COF的传感器将在其他真菌毒素的分析传感中得到越来越广泛的应用。

## 4 结论与展望

MOF和COF作为多孔有机框架材料的典型代表,因具有高比表面积、孔径可调节、活性位点分布均匀、结构可修饰等优异性能,为真菌毒素的分析提供了更多的研究方向。目前MOF和COF在真菌毒素中的应用多集中在食品污染方面,而对于中药材中真菌毒素的应用还有待挖掘。基于MOF和COF的电化学传感器在一定程度上满足了真菌毒素即时检测的需要,但用于真菌毒素的便携式荧光传感平台的研究还未见报道,有待进一步的探索。另外,虽然基于MOF或COF设计的材料已用于OTA、AFs等真菌毒素的分析,但同时富集、检测多种真菌毒素的报道有限。相信在不久的将来,以MOF和COF为代表的多孔有机框架材料在真菌毒素的分析中会发挥更大的作用。
